# Diplomatic response to global health challenges in recognizing patient needs: A qualitative interview study

**DOI:** 10.3389/fpubh.2023.1164940

**Published:** 2023-04-13

**Authors:** Jasna Karačić Zanetti, Matthew Brown, Marin Viđak, Ana Marušić

**Affiliations:** ^1^International Council of the Patient Ombudsman, Health Diplomacy Unit, Bruxelles, Belgium; ^2^University of Zagreb, Zagreb, Croatia; ^3^Global Health Policy Institute, University of California, San Diego, San Diego, CA, United States; ^4^Department of Research in Biomedicine and Health, Center for Evidence-Based Medicine, University of Split School of Medicine, Split, Croatia

**Keywords:** global health diplomacy, foreign affairs, health attachés, health policy, patient rights, COVID-19

## Abstract

**Background:**

Global health diplomacy is the applied practice of foreign affairs to further national goals that focus on health issues requiring international cooperation and collective action. We aimed to determine how international diplomats and health policy-related professionals in the EU understand the concept of health diplomacy, which impacts both diplomatic relations as well as patients' rights.

**Methods:**

In a qualitative interview study, we used a heterogeneous stratified purposeful sampling to reach participants from different countries and different practitioners from the Pyramid of Health Diplomacy: core, multi-stakeholder, and informal. Reflexive thematic analysis was used to identify the main themes.

**Findings:**

We contacted 131 practitioners of GHD, of which 37 responded, and nine agreed to be interviewed. From 11 interview questions, four main themes emerged from the analysis of the individual interview. The participants reported limited knowledge about the definition of GHD but also that they engaged in daily activities and decisions of inter-governmental bodies. They were not aware of existing special education and training for health attachés and made suggestions for improving the field and practice of GHD. They were not fully familiar with the European Charter of Patients' Rights. There was a consensus from all participants that patient rights need to improve as a fundamental right. They stressed the fact that the hospital lockdown and the right access to healthcare were impaired during the COVID pandemic.

**Interpretation:**

The role of health diplomacy in linking public health and foreign affairs is key to respecting patients' rights. Health over other interests is becoming an increasingly critical element in foreign policy. Establishing a clear career path for health attachés is necessary to foster effective global health agreements and coordination across countries.

## 1. Introduction

Global health diplomacy (GHD) is an interdisciplinary field of study that involves the integration of global health and foreign affairs. It aims to improve international relations while addressing the health needs of communities across national borders (1). The COVID-19 pandemic has highlighted the importance of GHD as policymakers seek to mitigate the impact of the pandemic and other public health threats (2). GHD is an emerging field that draws on the complementary expertise of global health and foreign affairs to address global health challenges and improve international relations (3).

The practice of GHD helps guide governments and non-state actors to coordinate global health action across borders to improve health, by bringing together these respective disciplines, including public health, which draw on skills in epidemiology, biostatistics, and scientific communication, as well as international affairs, which draws on skills in diplomacy, as well as e-governance, law, economics, trade policy, and more (4). Given the increasing need to mobilize disparate global health stakeholders, coupled with the need to design more complex public health partnerships to tackle health issues of international concern, effective and timely cooperation among state actors is critical (5). This is particularly relevant as health issues cross national boundaries and require global agreements to address and mitigate their impact on the health of communities more effectively (6). The relationship between health and foreign policy is complex and interdependent, highlighting the need for practitioners in both fields to critically evaluate the acquisition of skills, strengthening of competencies, and utilization of necessary tools to achieve their respective objectives. This requires a comprehensive and collaborative approach to identify and address the challenges faced by both communities of practice.

While there is a lot of academic activity surrounding the field of GHD, there remains a vague set of definitions and concepts that define its practice (2), especially in relation to the patient's rights, which together seek to render the fundamental rights appropriate to the current transitory situation in health services. Due to this lack of definitional clarity, the impact of the global political environment on health remains ineffective for patients' rights (7).

The COVID-19 pandemic is an example of why the practice of health diplomacy is so critical to stemming the spread of infection and disease, needing the successful application of skills in global health and disease control as well as diplomacy in foreign affairs, to coordinate with state and non-state actors, to provide rapid responses to the crisis, and join multiple nations together to stop the spread and mitigate the impact of the infection as it leaps across national borders around the world (8). The COVID-19 pandemic also illustrates the importance of collective action in global health as well, as the infection transcends national boundaries and necessitates governments to coordinate their response with neighboring countries while continuing to serve their respective populations (3). The pandemic has strained diplomatic relations among nations as issues around trade and transport of medicines, diagnostic tests, and critical hospital supplies and equipment needed to respond to the virus SARS-CoV-2 become increasingly scarce.

Amidst the COVID-19 pandemic, the practice of global health diplomacy (GHD) can serve as a valuable tool for countries seeking to identify joint interests, expand areas of agreement, and mobilize effective action on matters of common concern. Specifically, GHD can facilitate collaboration on critical issues such as access to health security, promotion of public health, disease control, and equitable access to essential medicines and technologies. By engaging in GHD practices, countries can work together to identify shared priorities and solutions, foster international cooperation, and ultimately contribute to improved global health outcomes (9).

In the European Union, each member state of the European Union has a Permanent Representation in Bruxelles, with members of diplomatic missions, who are charged with representing their respective governments (and Ministries) to the various working groups within the Council of the European Union, the third of the seven institutions of the European Union as listed in the Treaty on European Union (10). The Working Party on Public Health deals with public health issues and holds strategic discussions on common health-related issues, including addressing patients' rights in cross-border healthcare. The European Centre for Disease Prevention and Control (ECDC) mandate includes addressing issues related to cross-border threats to health, and the regulation of tobacco, organs, blood, and issues (11). The working party prepares the Employment, Social Policy, Health, and Consumer Affairs Council configuration (EPSCO) and, as appropriate, helps prepare EU positions in the international fora (such as within the multilateral health institutions, like the WHO).

The members of the European Commission and other subject matter experts are also invited to participate in these respective EU Working Groups. These meetings also support non-governmental professionals and other relevant representatives to participate in discussions at side events. Health policy and national reactions are delegated to a small group of health attachés, defined as a diplomat who collects, analyzes, and acts on information concerning health in a foreign country or countries and provides critical links between public health and foreign affairs stakeholders.

The present study seeks to gain insight into how global health diplomats perceive and navigate the intersection of patient rights and the practice of global health diplomacy (GHD). Specifically, this research aims to understand how international diplomats and health policy professionals in the European Union perceive the concept of health diplomacy, particularly in the context of patient rights during pandemics.

This study aimed to identify and understand key current and future changes affecting global public health and its impact on the patient's rights, to assess the impact of the COVID-19 pandemic on GHD, and to explore how GHD can be improved and be prepared for future pandemics.

## 2. Methods

### 2.1. Study design

Our study aimed to explore personal perceptions of health diplomacy and patients' rights of diplomats and health policy-related professionals in the EU as well as their understanding of patients' rights. We used a semi-structured qualitative interview approach, which enables discussion and construction of novel ideas. We followed the consolidated criteria for reporting qualitative research (COREQ) checklist for reporting the findings (12).

### 2.2. Participant selection

In this qualitative interview study, we enrolled stakeholders from different areas of health diplomacy. To ensure the representation of all relevant stakeholders, we used the global health diplomacy Pyramid model (13). Global health diplomacy pyramid consists of three categories of stakeholders in GHD, emphasizing the multi-disciplinary nature of GHD. Those are as follows: (1) Health attachés in the Council of the European Union (e.g., diplomats, core category of the GHD pyramid), (2) health policy-related professionals (e.g., government agencies, multi-stakeholder category of the GHD pyramid); and (3) informal participants in GHD (e.g., host county officials, NGOs, and universities and private enterprises, informal category of the GHD pyramid). We used a heterogeneous stratified purposeful sampling to reach participants from different countries (including different geographical parts of Europe), diplomatic or governance roles, international organizations, and the private sector.

**Figure 1:**
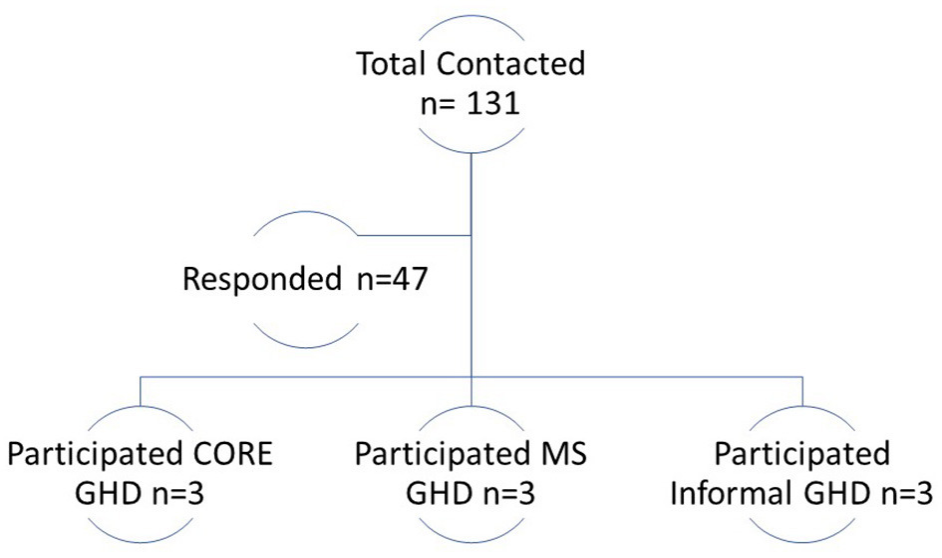
Participants included in the interviews that met inclusion criteria divided according to the Pyramid of Global Health Diplomacy. CORE: diplomatic professionals; health attachés and represent coordination focal points. MS: government agencies or entities of the federal government or a state or local government. Informal: members of host country officials, NGOs, universities, and private enterprises.

We emailed 33 health attachés (HA) from European countries' representatives delegated in mandate to the European Union (Figure 1). All accredited foreign diplomats are listed on a “Diplomatic List,” on their publicly available institutional web page. This list includes the individual's name and address, diplomatic rank, and formal title, which gives a brief indication of the diplomat's area of specialization and function. To identify additional participants, publicly available staff listings, panel and political summit programs dealing with global health diplomacy had been screened, and 98 health-policy-related professionals, private enterprise employees, and NGO representatives were contacted to participate in the study.

One HA agreed to participate in the study and two HAs refused to participate as they did not have the permission of their ambassador. There was no answer from the others (*n* = 30), even after repeated letters of request for an interview. Eight health-policy-related professionals and other stakeholders accepted the invitation to participate in the study, and the total number of participants was nine. Participants came from the following European Union countries: Sweden, Greece, Italy, Spain, Italy, Belgium, Netherlands, and Finland. One participant was from Switzerland, which belongs to the European Economic Area. There were three HA, three participants from government agencies (kom), and three from non-government and international organizations. There were six male and three female participants. All participants had a minimum of a university master's degree, and one had a doctoral degree. We did not receive age data from the three participants. The median age of those who provided age data was 41.5 years (interquartile range 36.5–49.5). The median of the years of experience in global health diplomacy was 7 (interquartile range 5.5–9).

### 2.3. Setting and data collection

The study was conducted online *via* Microsoft Teams from September 2020 to January 2021. Microsoft Teams is an online platform used to organize meetings and video calls and is safe and GDPR-compliant when used by countries in the European Union (11). During the interviews, only one researcher (JK) and the participant were present in the video call. Participants were given instructions to find a private and quiet environment to ensure privacy during the video call. The interviewer (JK) was a doctoral student at the time of the interviews, studying health diplomacy. Three participants had previously met and known the interviewer from EU meetings related to health and patient rights.

The questions to guide the interviews were developed previously, with an expert who had previous experience in qualitative research (MV) and were updated to match the current situation with the COVID-19 pandemic. We performed a total of nine interviews, each lasting from 35 to 60 min. Interviews were held in English and recorded. Field notes were not made during the interview and neither transcripts nor full texts were returned to participants for comments or corrections, nor were any repeated interviews carried out. Before the interview, participants completed a short questionnaire on socio-demographic data (gender, age, level of education, etc) and signed the informed consent. An example of a questionnaire is available in the Supplementary material.

### 2.4. Data analysis

Transcripts were analyzed using Braun and Clarke's six-step approach to reflexive thematic analysis (14). Reflexive thematic analysis is a flexible and adaptable method that can be used with a variety of qualitative data types and is used in the analysis of complex social phenomena. Following the familiarization with the data through transcription, reading, and re-reading, data were coded by JK using an inductive approach, followed by the development of subthemes for the interview questions. This was then discussed with all the authors. The code and data saturation approach were not used in this study as these are not used in the reflexive thematic analysis (12). We analyzed the transcripts using the NVivo computer program (NVivo Qualitative Data Analysis Software Version 12, QSR International, Australia).

### 2.5. Ethical approval and participants' protection

All participants received and signed the informed consent form prior to participating in the interviews. Transcripts were anonymized to preserve the identity of participants by JK and checked for accuracy by MV. Interviews were held after obtaining institutional ethical approval from the University of Split School of Medicine (IP-411 2014-09-7672). All data material is stored encrypted and safely at SharePoint, a web-based collaborative and GDPR-compliant platform, for 5 years after the last publication of the study. SharePoint is administered by scientists from the ProDeM project at the University of Split School of Medicine.

## 3. Results

Four main themes emerged from the data: (1) different perceptions and understanding of global health diplomacy, (2) the COVID-19 pandemic as a critical challenge of global health diplomacy, (3) patients' rights are a key task of the GHD, and (4) improvements in the GHD.

### 3.1. Perceptions and understanding of GHD

The participants recognized the importance of pursuing work in the area of GHD, particularly in collaboration with member states and other stakeholders in the health sector. This indicates a willingness to engage with the issue and work toward solutions that could have positive impacts on global health outcomes. In addition to the participants' limited knowledge about the definition of GHD, their responses also indicated a lack of familiarity with the topic.

“*GHD is where we're negotiating the issues of global health”* (*P1, Core)*.

“*Health diplomacy should be based on the solidarity between member states, solidarity between healthcare authorities or public health organizations, empathy on what healthcare and social care workers dealing with right now we're talking about.” (P6, Core)*.

The participants highlighted that GHD and diplomatic relations are the responsibility of the Ministry of foreign affairs and viewed GHD as a potential authority.

“*Most everything is interacting in various forms with both, my counterparts from the other member states, but also other institutions. And also I mean, from outside, the interest-oriented industry.” (P1, Core)*.

Not all participants stated that their career choice was planned. For some of them, it was a general interest to work with the EU, but there was not too much offer in the health-related area. One participant cited that the decision came from previous voluntary work, and one participant declared that it was an accident.

“*I was very young. I was very attracted by knowledge, by understanding things. I was very curious, and I wanted also the things that I thought I could maybe discover that could make a change in people's lives. Right. Positive change.” (P6, Core)*.

The participants reported that they engaged in daily activities of such debates and decisions of inter-governmental bodies; working to strengthen countries' effectiveness and leadership role in health; creating and sustaining effective networks and coalitions with the relevant partners, contributing to a coherent and effective system at global, regional, and country levels. The participants stated that they interact with all key decision-making bodies to make a wide range of recommendations on policy issues, inter-agency and inter-governmental collaboration and activities, relations with NGOs, and the media.

“*That means for us […] collaboration within the European collaboration or even with the WHO, OECD, and we have other global and other international collaborations like the Global Digital Health Partnership or the Global Consortium for E-health Interoperability” (P6, Core)*.

“*It allows me, to be in contact with several stakeholders. So especially in the healthcare sector, you have so many different types of stakeholders, as some professional organizations to the International European Organization. I quite like it.” (P5, Multi-stakeholder)*.

The participants were not aware of existing special education and training for health attachés. Participants reported having received a general education in international relations, with limited exposure to healthcare-related topics in either the private or government sector.

“*If you referring to the University of Education, it was more general. It was not specific on health in any way.” (P5, Multi-stakeholder)*.

“*I had various types of courses, but not specifically on that topic.” (P8, Multi-stakeholder)*.

All participants highlighted the need for specific training for practicing global health diplomacy and serving in a diplomatic mission.

“*I've never done health diplomacy training, that's for the director level. I would like it very much but I had not done it. And so most of what I know is from my own experience and of course from learning as I go along.” (P5, Multi-stakeholder)*.

“*I'm not aware of training opportunities in healthcare. I wasn't even aware about the notion of health diplomacy, this does not mean that they do not exist it is simply that I'm not aware of.” (P1, Core)*.

The participants highlight the importance of skills, communication, leadership, conflict resolution, and emotional intelligence to navigate professional interactions in GHD.

“*Well, first of all, I think that you have to understand how things work at the global level and also, you know in your level. You have to be connected.” (P2, Core)*.

### 3.2. Pandemic is a critical challenge for the GHD

Participants expressed a consensus that the current COVID-19 pandemic has highlighted the urgency for effective global health diplomacy to tackle the diverse health challenges faced by each nation. The Commission's initiative to establish a centralized approach for procuring supplies and promoting vaccine development within the EU was cited by multiple participants as a prime example of how GHD can facilitate better health outcomes.

“*We're collaborating on a range of issues, both on the EU level and in other aspects, on, of course, other organizations (…) So I would say the whole range of collaborations that are going on a daily basis.” (P2, Core)*.

“*We need to learn how to how to implement public health measures not because of COVID-19 but because we have to do it.” (P6, Core)*.

The participants emphasized that the pandemic represents a global crisis that requires unforeseen diplomatic relations between countries. Participant discusses what we can learn from the failure of diplomacy to prevent, halt, and wrap up the situation and want to know the reasons for the failure of diplomacy.

“*Global crisis can be addressed through bilateral cooperation, through transnational cooperation, through multilateral cooperation. We need to set views, we need to change, we need to adopt good practices. We need to find the golden standards so we can learn the lesson from this pandemic and to march toward the future by being wiser.” (P3, Informal)*.

“*I think that there is still a great deal of work to do there. But I'm also thinking about the relation between the developed and countries under development, especially Africa…” (P1, Core)*.

The participants criticized the weak evaluation process and decision-making of the relevant international health organizations. Participants criticized the WHO and other international agencies for being too late in the development of protocols while failing to provide evidence-based and clear instructions to the national health authorities. Some participants provided explanations for the international health organization in the early phases of the pandemic.

“*I share the view that the WHO has not been up to the challenge, especially in the beginning.” (P2, Core)*.

“*The one thing that is criticized now is to what extent the reaction should have been earlier on when we started knowing the cases in China. We should have really acted at the time.” (P2, Core)*.

“*I believe that government, especially also in Europe, didn't take all the preparedness policies into action to prepare for a pandemic at the beginning. I feel that the European response was a little bit late.” (P9, Informal)*.

“*Health has the problem that nobody is in charge, so there's no way can do it right, because, when something good happens, no reactions and if something bad happens, then i is failed to coordinate or lead or provide resources. So someone is always at the wrong end of the stick. And that's currently unavoidable.” (P4, Informal)*.

### 3.3. Patient's right is the key task of the GHD

There was a consensus from all participants that patient rights need to improve as a fundamental right first. It can be improved by changing the culture of medical professionals. They need to be aware of these issues.

“*Human rights and patient rights are fundamental. I believe that right now there is also an issue on how to make the patient's also actor not only subject of medical issues, also at the global level. I believe that this initiative taking into consideration and also the legal framework of human rights it could be taking into account.”* (*P2, Core)*.

Participants identified the relevance and importance of the patient's rights to the GHD, and it is described as fundamental.

“*I think that there is a kind of improvement going on, I'm here in Brussels for 21 years now, but when I arrived here, I think that the real notion of patient rights was not really in the air, and over time, I saw really important change happening, especially e in the hospitals.” (P5, Multi-stakeholder)*.

“*…patient's rights are always relevant regardless of exactly the setting. It is something that will, of course, be present in various contexts that negotiating.” (P5, Multi-stakeholder)*.

“*We need it patient rights more crucial, more important in the decision-making process, patients, will, desires, and perceptions are in the core. Placing the core in our field of academic background.” (P6, Core)*.

The level of awareness among the participants regarding the European Charter of Patients' Rights was limited, as none of them reported a comprehensive understanding of the document.

“*Well, I'm not familiar with that, I've read many papers on patient's rights have also participated in the national congresses where the patient's community, very crucial and beneficial contribution to Congress's activities. But I'm not aware of what you say.”* (*P1, Core)*.

“*I don't know. I know the Charter of Fundamental Rights, but not this one.”* (*P2, Core)*.

Participants recognized patients' rights during the COVID-19 pandemic as particularly important, as the right to access healthcare across the globe was impaired. Participants identified the COVID-19 pandemic as a huge challenge to obtain rights in emergencies.

“*…it's a challenge for the health care system to try maintaining the regular production, of course. And obviously, there have been situations where, for example, nonurgent procedures to be postponed. Obviously, it may effect on the access when it comes to other treatments or proceedings.” (P2, Core)*.

“*I do not think any health system is capable of fully taking on the burden that this pandemic brings and the regular health care burden.”* (*P1, Core)*.

### 3.4. Future improvements are needed in the GHD

According to the participants, GHD should aim to develop comprehensive guidelines for addressing different health issues in diverse settings and provide relevant and up-to-date information to national health policymakers.

“*There are guideline recommendations and so on that can be both can be established in various levels.” (P6, Core)*.

“*I cannot say if this is an issue of protocol missing or it is more the kind of issue related to how the health care systems are organized, but for sure there has been really something missing there.” (P6, Core)*.

The participants resolutely believed that GHD as a field and practice would continue to expand and develop in importance in future and then we need to have diplomatic health professionals to be ready for future threats because the chance of a new pandemic wreaking world is quite high.

“*There are reflection processes already ongoing, both on a WHO level, but also the EU level. So it will be trying to figure trying to sort of find a good, good answer to those things will, of course, be important.” (P7, Multi-stakeholder)*.

Some participants stated that the cooperation between the scientific community and the healthcare community and the patients should be strengthened after this pandemic.

“*We will have to have a clear post-pandemic analysis. To really try to identify what was wrong and learn from are not errors, but let us say problems or things that we didn't do efficiently. We need to get rid of the politicization, that all the pandemic has had and define how general public, how professionals, how patients should be informed. I think that we need to do in these how you communicate things to the public.” (P2, Core)*.

All participants agreed that there is a need for broader engagement of the countries of the region in strengthening the interface of health and foreign policy, both within and across countries, with the support of WHO. The goals of the process of engagement need to be clear, and transparency and accountability need to be ensured.

“*Well, I think that we have been work with at the time, of course, is to make sure that we have an open and ongoing dialogue between the representation, the various ministries in the capital, and also the representation in Geneva and New York.” (P8, Multi-stakeholder)*.

## 4. Discussion

The result of our study highlights that little is known about the definition of GHD among all stakeholders. Global health needs global health diplomacy and the participants define multiple ways for the improvement of GHD and their practice, as health becomes an ever more critical element in foreign policy, security policy, and development strategies. An increasing number of health challenges as managing the COVID-19 pandemic can no longer be resolved at the technical level only, as health diplomacy takes place at many levels and involve a wide range of participants.

As the world has become more interconnected, the need for coordinated responses to share global public health threats has increased. A small but growing cadre of diplomats known as health attachés is key among the practitioners of global health diplomacy (GHD) who employ the tools of diplomacy and statecraft to bridge governments' public health and foreign policy objectives.

The participants' perspective shows that health is fundamental to economic and social wellbeing and recovery and also to national security and so the engagement has to be with multiple sectors, including leaders who make the decisions about resource allocations. The GHD core principle relies on the concept of bringing nations together in diplomatic missions to confront public health threats that all countries need to prepare for and has a vital role in sharing information (15).

The value of accessing the views of both diplomats and health-related professionals on problematic conceptual understanding of HD is now being recognized. Health attachés are key practitioners and can help facilitate a significant impact on the current and future path of GHD (16). As stated in the literature, the skills of diplomacy and negotiation, applied science, and intercultural competencies are key to educating health attachés. Negotiation skills are essential for health attachés to effectively communicate and collaborate with stakeholders at different levels, including government officials, NGOs, and healthcare providers. They must be able to identify common goals and interests and work toward mutually beneficial outcomes. Overall, these findings are in accordance with findings reported by Brown et al., in which the skills of diplomacy and negotiation, applied science, and intercultural competencies are key to educating health attachés. In addition, establishing a clear career path for health attachés is crucial for the future maturation of the profession and for fostering effective global health actions that align public health outcomes and foreign policy outcomes.

Another promising finding from our study was that the COVID-19 pandemic has demonstrated the importance of global health diplomacy (17). Despite the lessons that were to be learned from emergency preparedness and response to HIV and EBOLA (18) when SARS-CoV-2 unexpectedly hit the world, we identified important lessons that GHD can apply to help healthcare professionals in the COVID pandemic. Participants reported that the COVID-19 pandemic was a very difficult time for healthcare professionals who must balance the fear for their own safety by providing full care to their patients. They face ethical and moral dilemmas about limiting access to healthcare and the risks of COVID-19. They also had to choose between providing care or postponing an already scheduled diagnostic and therapeutic procedure, but they also had to obey the rules of health facilities. Most participants stressed the fact that healthcare professionals must always have national support and hospital support for decision-making. COVID-19 transcends national borders and governments and calls for global action to determine human health.

Another important finding from our study was the importance of patients' rights in global health crises. The COVID-19 pandemic is beyond national borders and governments and calls for global action to determine human health; however, understanding the needs of each individual patient locally leads to the best treatment process and establishes a stronger relationship of trust between patients and healthcare professionals. Our results indicate that understanding the needs of each patient locally brings together the best treatment outcome and establishes a stronger relationship of trust between patients and healthcare professionals. Trust, knowledge, regard, and loyalty are four elements that form the doctor–patient relationship and the nature of this relationship has an impact on patient outcomes (19). National strategies, policies, and diplomacy must be guided by the principle that only a well-informed patient can be involved in joint decision-making which will result in the best use of healthcare (20).

Health diplomacy had an indispensable role in the control of vaccine development and distribution. More activities such as COVID-19 Vaccines Global Access (COVAX) that guarantee vaccine equity should be implemented and should take into account to assist in distributing vaccines to lower-income countries. The global picture of access to COVID-19 vaccines was unacceptable that just one-fifth of people in low- and lower-middle-income countries had received their first dose of the vaccine, while in higher-income level countries, 80% had received a dose. Despite widespread calls by health actors for more effective coordination at the state level, formal communication mechanisms are often fragmented by diseases, sectors, or bureaucratic silos. Public health professionals can act without awareness of major diplomatic strategies (21, 22).

Health diplomacy can raise awareness that health is not just a national issue; it also includes different global and cross-border dimensions and can make a significant contribution to the action of public interest for global development and the wellbeing of people around the world (23). Health diplomacy is also a response to the fact that many of the health challenges of the twenty-first century will require solutions that will be more political than technical (24).

Based on our study, several recommendations can be put forward regarding diplomatic responses to global health threats:

1. Recognize that the necessary response to the growing incidence of global threats to health and violations of patients' rights, both nationally and internationally, includes improved governance of health systems.
2. The intensification of cross-border health threats arising from the increasing spread of globalization is such that it is not possible for any individual state or organization to solve the problems it faces on its own.
3. Evidence-based healthcare should be implemented into diplomatic work.

### 4.1. Limitations

The main weakness of our study was the limited sample of respondents. It was very difficult to get participants from diplomatic missions. As reported, this study focused only on the main practitioners of GHD, which is by definition very small and specific. However, the lack of a formal conceptual framework for the area of GHD and the undefined job description for a ‘health diplomat' require more information than those dealing with diplomacy in this area.

In addition, one of the limitations could be restraint in the answers to what they are allowed to say because the very role in state national activities requires a high degree of loyalty and the inability to give a different and personal opinion.

## 5. Conclusion

The role of health diplomacy is crucial for ensuring patient rights. Health is becoming an increasingly critical element in foreign policy and should imperatively acquire new skills for negotiating in favor of health over other interests in political security, development strategies, and health and foreign policymakers. Diplomacy and negotiation skills, applied sciences, and intercultural competencies are essential for the work of health attachés. Establishing a clear career path for health attachés is key to the future evolution of the profession and to achieving effective global health diplomacy that aligns public health and foreign policy. The adoption of a more all-encompassing view of the aims of health diplomacy and acceptance of the need to provide “empowering” explanations that encourage politicians to understand concepts of patients' needs could offer a way forward. The COVID-19 pandemic has demonstrated the vital importance of global solidarity to confront common public health warnings and showed the importance of global Health diplomacy with duties to protect health professionals so that they can protect their patients.

## Data availability statement

The original contributions presented in the study are included in the article/Supplementary material, further inquiries can be directed to the corresponding author.

## Ethics statement

All participants received and signed the informed consent form prior to participation in the interviews. Transcripts were anonymised to preserve the identity of participants by JK and checked for accuracy by MV. Transcripts were not returned to participants for corrections and comments. Before the interview, participants completed a short questionnaire on socio-demographic data (gender, age, level of education…). An example of a questionnaire is available in the Supplementary material. All interviews were held after obtaining institutional ethical approval from the University of Split School of Medicine (IP-411 2014-09-7672). All data material is stored encrypted and safely at SharePoint, a web-based collaborative and GDPR compliant platform, for 5 years after the last publication of the study. SharePoint is administered by the scientists from the ProDeM project at the University of Split School of Medicine.

## Author contributions

JK, MB, and AM designed the study. JK had full access to all the data in the present study. MV and JK interpreted the results. AM supervised data collection and analysis. All authors take responsibility for the accuracy of the data analysis. All authors contributed to the article and approved the submitted version.
