# Supplementary material for: Diplomatic response to global health challenges in recognizing patient needs: A qualitative interview study
Source: Front Public Health. 2023 Apr 13;11:1164940. doi: 10.3389/fpubh.2023.1164940 (PMC10136764; doi:10.3389/fpubh.2023.1164940)
Supplement: Supplementary file 1 [file Table_1.docx]

Supplementary Material

Diplomatic response to global health challenges in recognizing patient needs: a qualitative interview study

Jasna Karačić Zanetti *^1,2^, Matthew Brown ^3^, Marin Viđak ^4^, Ana Marušić ^4^

* Correspondence: Corresponding Author: jkaracic@unizg.hr

Appendix: Interview questions

| 1. What are your responsibilities and portfolio of health-related activities or topics? As there an official organizational structure within your Embassy for managing health issues? |
| --- |
| 2. What are the other organizations and entities you collaborate with related to global health projects or negotiations? Can you describe any relevant global health accomplishments or negotiations of which you have been part? |
| 3. Have you had any specific training in global health diplomacy? Are there options for the training programs in which you have participated to include health diplomacy training? What skills are important to have as practicing health diplomacy – as a health attaché? |
| 4. What is your definition of global health diplomacy? Do you think anything specific should be done to improve the practice of GHD? |
| 5. Are patients' rights relevant for GHD? In Your personal opinion, can patients' rights be improved? For example: The Universal Declaration of Human Rights Formalized in 1948, recognizes “the inherent dignity” and the “equal and unalienable rights what is owed to the patient as a human being and took shape in large part thanks to this understanding of the basic rights of the person by physicians and by the state. What is your experience? |
| 6. Are you familiar with The European Charter of 14 Patients' Rights launched in 2002? Are they addressed in your work or at diplomatic meetings? |
| 7. In your opinion, how well are patients in your country and in EU informed about their rights as patients? |
| 8. What do you think about patient rights in the COVID-19 pandemic? Were they affected during these preventive measures, which limited limiting access to healthcare? |
| 9. How global health diplomacy can help to professionals, who have to balance the fear about their own safety by providing full care to their patients? |
| 10. What do you think of the reaction of national and international health organizations to the COVID-19 pandemic? |
| 11. Does the COVID-19 pandemic represent a global crisis that requires unforeseen diplomatic relations between countries to respond more quickly to threatening pandemics in the future? How can we ensure that patients’ rights are respected? |
